# Case Report: Cryoanalgesia for intractable thoracic pain due to chest wall tumor

**DOI:** 10.3389/fonc.2025.1516805

**Published:** 2025-08-06

**Authors:** Thays Sellan Paim, Myrna Guernelli, Rafael Soares Simoneti, André Miotto

**Affiliations:** Department of Thoracic Surgery, Universidade Federal de São Paulo, São Paulo, Brazil

**Keywords:** thoracic surgery, carcinoid tumor, chest pain, cryoanalgesia, thoracic wall, case report

## Abstract

**Introduction:**

Postoperative pain in thoracic surgery is a major concern, often delaying recovery. Cryoanalgesia, using pressurized nitrous oxide, offers long-term pain relief by temporarily blocking peripheral nerve function. It takes effect within 72 hours, so an intercostal nerve block is used for immediate pain relief. Cryoanalgesia is effective for severe pain, with minimal side effects and no drug interactions.

**Case report:**

A 63-year-old man with severe thoracic pain, unresponsive to opioids, was diagnosed with a typical carcinoid tumor after a CT scan revealed lesions in his lung, thoracic wall, and adrenal gland. He underwent surgery for the thoracic lesions with cryoanalgesia, leading to excellent recovery and no pain. A month later, the adrenal lesion was removed laparoscopically. He remains pain-free and free of recurrence after one year, indicating successful treatment.

**Conclusion:**

Cryoanalgesia provides long-term relief for chest pain caused by tumors, with minimal side effects. It improves recovery and should be considered an essential part of pain management in thoracic surgery.

## 1 Introduction

Postoperative pain is a significant concern for patients undergoing thoracic surgery, often complicating recovery and prolonging hospital stays (1). To address this challenge, innovative pain management techniques such as cryoanalgesia have been developed, offering a promising alternative for alleviating postoperative pain (2).

Treatment with cryoanalgesia includes application of pressurized nitrous oxide for neurolysis of the axon and myelin sheath to specific nerves to a reversibly inhibiting peripheral nerve function, thereby temporarily blocking pain conduction (2–4). The goal of the method is to provide long-term pain relief, and it is even used for intractable pain (5). The effect of the procedure begins in approximately 72 hours. For this reason, in thoracic surgery, intercostal nerve block is also commonly performed during surgery to reduce pain until the cryoanalgesia takes effect. Furthermore, they have no drug interactions or contraindications, and their adverse effects are minimal (3, 4).

The use of cryoanalgesia in thoracic surgery is still a relatively new and evolving technique, showing great promise in the management of postoperative pain. Although its application in other fields, such as chronic spinal pain, is more established, its introduction into thoracic procedures opens up exciting possibilities for enhancing patient recovery. As research continues, cryoanalgesia may become a standard approach in thoracic surgery, offering a safer and more effective alternative for pain management.

## 2 Case report

A 63-year-old male patient, person of Afro-Latin descent, presented with intractable thoracic pain, which did not improve even with opioid analgesics, initially using 30 mg codeine every 6 hours, and with no pain relief, morphine was introduced. There were no abnormalities on physical examination. She had no significant personal or family medical history and no prior smoking history. During the etiological investigation, chest and abdominal computed tomography scan revealed a pulmonary nodule and a lesion in the posterior thoracic wall, with rib invasion, as well as another lesion in the right adrenal gland.

**Figure 1:**
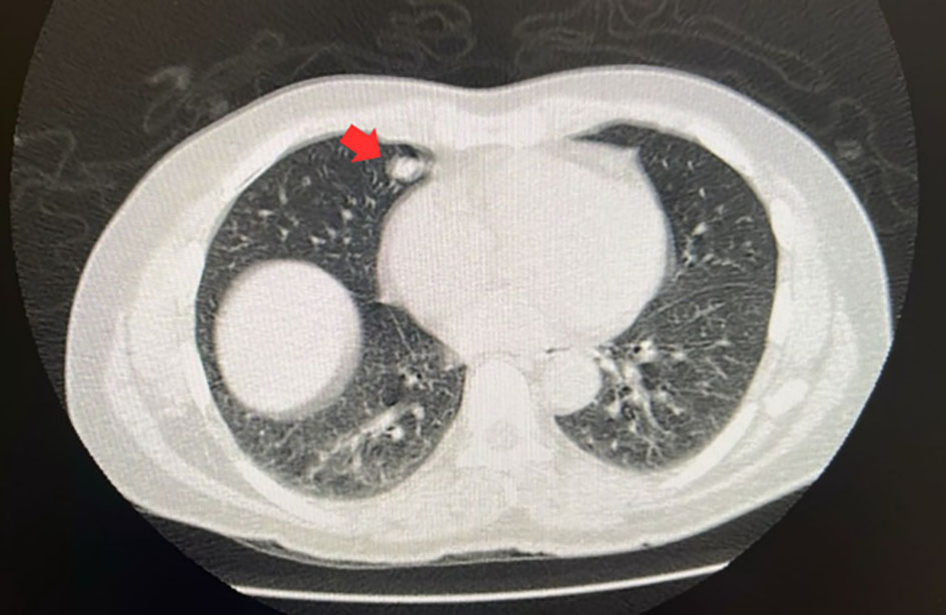
The red arrow indicates the pulmonary nodule.

**Figure 2:**
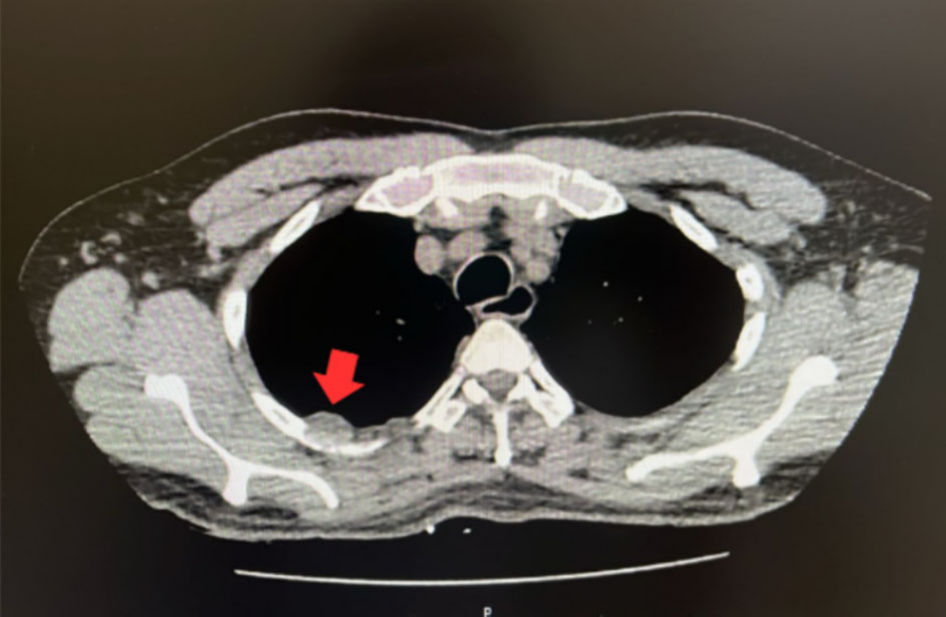
The red arrow indicates the posterior thoracic wall nodule.

Preoperative exam CT images: (**Figures 1** and **2**):

The biopsy of both thoracic lesions, performed by CT-guided transthoracic technique, showed a typical carcinoid tumor. Thus, a diagnosis of typical carcinoid tumor associated with chronic pain was made.

**Figure 3:**
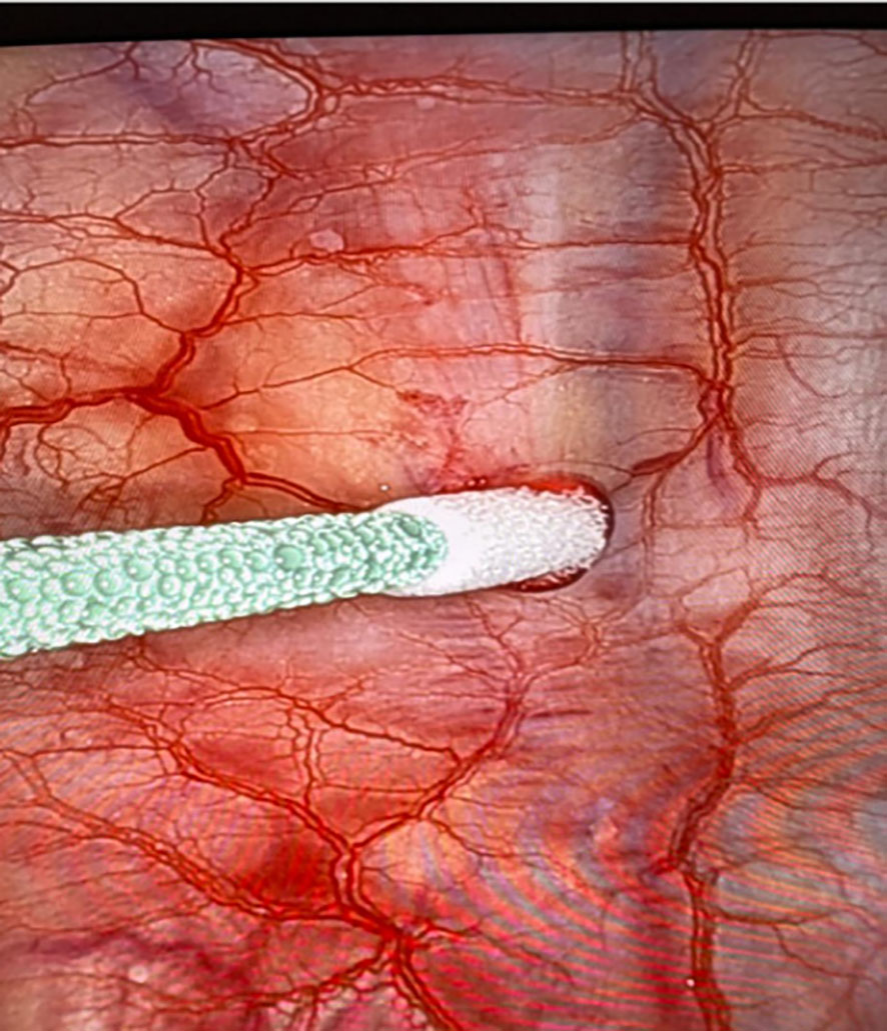
Cryoanalgesia procedure.

In the surgical approach, a pulmonary segmentectomy was performed by videothoracoscopy. Chest wall resection, including the posterior lesion, was performed at the same surgical time, along with cryoanalgesia (Metrum Cryoflex - Poland), aiming to promote better postoperative recovery and relief of thoracic pain. The procedure involved opening the parietal pleura and placing a probe in the intercostal space with light pressure. A freezing cycle of 2 minutes at -78°C was applied, with the temperature maintained constant throughout the cycle (**Figure 3**). Although it is not possible to quantify the amount of carbon dioxide used, the temperature remained stable during the process.

The patient had an excellent recovery, with no pain from the immediate postoperative period, and was discharged 3 days after surgery. The histopathological examination of both lesions confirmed the diagnosis of a typical carcinoid tumor. Additionally, in the month following the thoracic surgery, resection of the adrenal nodule was performed by laparoscopy.

The patient is currently under follow-up and has been free of recurrence or metastasis for over a year, confirming the oncologically correct treatment. The patient has remained pain free, showing a good prognosis as the lesions were completely resected.

## 3 Discussion

### 3.1 Effective pain management

The primary benefit of cryoanalgesia is its efficacy in providing significant pain relief. By applying pressurized nitrous oxide to achieve neurolysis of the axon and myelin sheath, cryoanalgesia temporarily inhibits peripheral nerve function, effectively blocking pain conduction. This technique has been proven particularly effective in patients with intractable pain, as seen in cases where opioid analgesics, including morphine, fail to provide relief. For example, cryoanalgesia successfully alleviated the pain of a patient who had been unresponsive to opioid treatments, facilitating a smoother and more comfortable postoperative recovery. Additionally, this method is aligned with findings from studies showing long-term pain relief, particularly in patients with refractory chest wall symptoms, where conventional therapies had failed.

Cryoneurolysis is associated with minimal risk, as it preserves vital structures like vessels and bone, preventing complications such as neuroma or pseudohernia formation. Furthermore, cryoanalgesia provides an effective alternative for patients with severe rib fractures, reducing narcotic use and offering durable pain control. Although neuralgia has been reported as a postoperative complication in up to 20%–30% of patients in some studies, this has not been consistently observed in all reviews. Despite these isolated complications, cryoanalgesia remains a low-risk modality, supporting its use for pain management in challenging clinical situations (6, 7). A recent systematic review found that cryoablation of intercostal nerves significantly reduced hospital stays compared to thoracic epidural analgesia, underscoring its benefits in pain management after procedures like the Nuss procedure, though some studies did find heterogeneity in the results due to variations in additional pain therapies used (8, 9).

Cryoanalgesia is effective in reducing acute pain, opioid requirements, and length of hospital stay, especially in pediatric patients, who are at lower risk of developing postoperative neuropathic pain (10, 11). However, adults undergoing intercostal cryoanalgesia have a higher risk of developing chronic neuropathic pain and prolonged numbness, with some studies reporting neuropathic pain incidence rates ranging from 20% to 30% (10, 12). Compared to epidural analgesia, cryoanalgesia may be associated with higher intensity of neuropathic pain, allodynia, and a negative impact on daily activities, particularly in long-term follow-up. Despite these risks, the technique is considered safe, minimally invasive, and effective for managing both acute and chronic pain, representing a relevant alternative in scenarios of difficult analgesic control. However, it is important to acknowledge the potential contribution of placebo effects in the perception of pain relief, particularly in single-case reports or studies lacking control groups. The subjective nature of pain and the strong influence of patient expectations may lead to an overestimation of the true efficacy of cryoanalgesia in some contexts. Differentiating between genuine physiological effects and placebo-induced responses remains a challenge, reinforcing the need for controlled studies to more accurately assess outcomes (13, 14). The heterogeneity of studies, differences in technique, and the specific nerves treated make direct comparisons challenging and hinder a clear definition of the actual risk of neuropathic pain (15).

### 3.2 Long-term pain relief

Cryoanalgesia is designed to offer long-term pain relief, which is crucial for patients undergoing major surgeries such as thoracic operations. The procedure’s effects typically begin around 72 hours post-application, during which an intercostal nerve block can be used to manage pain until cryoanalgesia takes full effect. This dual approach ensures continuous pain management, as evidenced by the patient’s immediate postoperative pain relief and ongoing pain-free status over a year post-surgery. In trauma patients, such as those with severe rib fractures, cryoanalgesia has demonstrated the potential for long-term pain control, addressing the shortcomings of traditional pain management methods like narcotics, which carry the risks of oversedation and respiratory depression. Cryoneurolysis of intercostal nerves after rib fractures, for example, provides a minimally invasive alternative that offers durable pain control and can be applied alongside other treatments (7, 8).

### 3.3 Minimal adverse effects and contraindications

Another significant advantage of cryoanalgesia is its minimal adverse effects and lack of drug interactions or contraindications. This safety profile makes it an attractive option for patients who may have contraindications to other forms of pain management or those who experience significant side effects from medications (16). Cryoanalgesia has been reported to reduce narcotic use and shorten hospital stays, contributing to enhanced recovery and improved patient outcomes. A study on pediatric patients undergoing thoracotomy for pectus excavatum demonstrated that cryoanalgesia was associated with reduced postoperative complications, such as pneumothorax and neuralgia, while maintaining low complication rates overall (17, 18).

Notably, a meta-analysis on cryoablation of intercostal nerves also found a significant reduction in hospital stay, with cryoablation patients being discharged 2.91 days earlier than those receiving thoracic epidural analgesia. This was largely attributed to the reduced need for additional pain therapies and decreased opioid usage (8). Cryoanalgesia’s ability to provide long-lasting pain relief without the need for frequent follow-ups or complex procedures further adds to its appeal in clinical settings.

### 3.4 Enhanced recovery and reduced hospital stay

The integration of cryoanalgesia in the surgical treatment plan not only managed the patient’s pain but also contributed to a swift recovery and reduced hospital stay. The patient was discharged only three days post-surgery, indicating that effective pain control facilitated early mobilization and reduced postoperative complications, which are often exacerbated by pain. This is consistent with other studies, which also demonstrated shorter hospital stays and reduced opioid use in patients following cryoanalgesia (17, 18). Cryoanalgesia’s ability to enhance recovery is especially beneficial in trauma cases, where pain management can play a key role in minimizing the need for prolonged hospitalization. Moreover, studies on trauma patients, particularly those with rib fractures, have demonstrated the benefits of cryoanalgesia in reducing inpatient narcotic use and shortening hospital stays (8, 9).

### 3.5 Oncological outcomes

While the primary focus here is on pain management, it’s notable that the patient also achieved excellent oncological outcomes. The comprehensive surgical approach, combined with effective postoperative pain management, likely contributed to the patient’s ability to undergo subsequent necessary treatments, such as the resection of the adrenal nodule, and maintain a pain-free status during follow-up. This broad treatment plan has kept the patient free from recurrence or metastasis for over a year, demonstrating that effective pain management can support overall treatment success. This principle is reflected in other studies, where cryoanalgesia was used in pediatric patients undergoing thoracotomy, further underscoring its role in not only pain control but also in enabling continued oncological care (19). In fact, some studies suggest that cryoanalgesia, by reducing opioid dependence, may improve patient mobility and outcomes in post-surgical cancer care (8, 9).

### 3.6 Carcinoid tumor

Carcinoid tumors are rare neuroendocrine pulmonary neoplasms, typically characterized by slow growth and an indolent clinical course. The primary curative treatment is complete surgical resection, making surgery the preferred approach for optimal outcomes. Pulmonary carcinoid tumors are classified as typical or atypical. Typical carcinoids are more common, grow slowly, and rarely metastasize, whereas atypical carcinoids are less frequent, exhibit a higher growth rate, and have an increased metastatic potential. Although the reported case involves a typical carcinoid tumor, its behavior is unusual for this subtype, as it demonstrates rapid growth and metastatic spread.

### 3.7 Strengths and limitations associated with this case report

Cryoanalgesia is a prolonged analgesia method with few contraindications, allowing its use to be extended to oncological cases. The main limitation of the method is its limited accessibility and high cost. Moreover, while cryoanalgesia can decrease inpatient narcotic use and shorten hospital length of stay, there is still a need for further studies, particularly in trauma patients. Current evidence for its use in traumatic rib fractures remains limited, and additional prospective randomized controlled trials are necessary to fully evaluate its efficacy and long-term benefits. A recent meta-analysis of cryoablation in Nuss procedure patients corroborates these findings, suggesting a shorter hospitalization and reduced opioid use, though its effectiveness remains variable across studies (20). The reported case in this paper highlights the significant role of cryoanalgesia in providing rapid and lasting pain relief, establishing its importance in both oncological and trauma settings.

## 4 Conclusion

Cryoanalgesia offers a promising solution for managing intractable chest pain associated with chest wall tumors, providing significant, long-term pain relief with minimal adverse effects. Its successful application in this case underscores its potential to enhance postoperative recovery, reduce hospital stays, and contribute to better overall treatment outcomes. As such, cryoanalgesia should be considered a valuable component in the multimodal approach to pain management in thoracic surgery. Currently, the biggest challenge for the dissemination of this technique is access to cryoanalgesia, which remains limited, primarily due to costs.

## Data Availability

The original contributions presented in the study are included in the article/supplementary material. Further inquiries can be directed to the corresponding author.
